# FCγ Chimeric Receptor-Engineered T Cells: Methodology, Advantages, Limitations, and Clinical Relevance

**DOI:** 10.3389/fimmu.2017.00457

**Published:** 2017-04-27

**Authors:** Sara Caratelli, Tommaso Sconocchia, Roberto Arriga, Andrea Coppola, Giulia Lanzilli, Davide Lauro, Adriano Venditti, Maria Ilaria Del Principe, Francesco Buccisano, Luca Maurillo, Soldano Ferrone, Giuseppe Sconocchia

**Affiliations:** ^1^Institute of Translational Pharmacology, CNR, Rome, Italy; ^2^Department of Systems Medicine, University of Rome “Tor Vergata”, Rome, Italy; ^3^Department of Biomedicine and Prevention, University of Rome “Tor Vergata”, Rome, Italy; ^4^Departments of Surgery and of Orthopedic Surgery, Massachusetts General Hospital, Harvard Medical School, Boston, MA, USA

**Keywords:** antitumor activity, chimeric antigen receptor T cells, CD16-CR T cells, CRC, Fc gamma chimeric receptor, hematologic malignancies, immunotherapy, solid tumor

## Abstract

For many years, disappointing results have been generated by many investigations, which have utilized a variety of immunologic strategies to enhance the ability of a patient’s immune system to recognize and eliminate malignant cells. However, in recent years, immunotherapy has been used successfully for the treatment of hematologic and solid malignancies. The impressive clinical responses observed in many types of cancer have convinced even the most skeptical clinical oncologists that a patient’s immune system can recognize and reject his tumor if appropriate strategies are implemented. The success immunotherapy is due to the development of at least three therapeutic strategies. They include tumor-associated antigen (TAA)-specific monoclonal antibodies (mAbs), T cell checkpoint blockade, and TAA-specific chimeric antigen receptors (CARs) T cell-based immunotherapy. However, the full realization of the therapeutic potential of these approaches requires the development of strategies to counteract and overcome some limitations. They include off-target toxicity and mechanisms of cancer immune evasion, which obstacle the successful clinical application of mAbs and CAR T cell-based immunotherapies. Thus, we and others have developed the Fc gamma chimeric receptors (Fcγ-CRs)-based strategy. Like CARs, Fcγ-CRs are composed of an intracellular tail resulting from the fusion of a co-stimulatory molecule with the T cell receptor ζ chain. In contrast, the extracellular CAR single-chain variable fragment (scFv), which recognizes the targeted TAA, has been replaced with the extracellular portion of the FcγRIIIA (CD16). Fcγ-CR T cells have a few intriguing features. First, given in combination with mAbs, Fcγ-CR T cells mediate anticancer activity *in vitro* and *in vivo* by an antibody-mediated cellular cytotoxicity mechanism. Second, CD16-CR T cells can target multiple cancer types provided that TAA-specific mAbs with the appropriate specificity are available. Third, the off-target effect of CD16-CR T cells may be controlled by withdrawing the mAb administration. The goal of this manuscript was threefold. First, we review the current state-of-the-art of preclinical CD16-CR T cell technology. Second, we describe its *in vitro* and *in vivo* antitumor activity. Finally, we compare the advantages and limitations of the CD16-CR T cell technology with those of CAR T cell methodology.

In 1989, Gross and colleagues introduced the concept of engineering T cells with chimeric receptors capable of overcoming the major histocompatibility complex restriction, laying the groundwork to generate a powerful tool for targeted cancer immunotherapy (1). The chimeric antigen receptors (CARs) are molecules capable of redirecting cytotoxic T cells (CTLs) against a tumor-associated antigen (TAA) expressed on the surface of malignant cells. A CAR typically consists of a single-chain variable fragment (scFv) derived from the antigen binding region of a TAA-specific mAb linked to the T cell receptor (TCR)-associated CD3ζ-chain signaling domain (2). This structure refers to the first generation of CAR. *In vitro* and *in vivo* studies, performed using CD3ζ-CARs, showed promising results demonstrating an efficient tumor cell elimination. However, the following clinical trials failed to confirm the first generation CAR-T cell therapeutic efficacy, although a first-generation CAR targeting GD2 induced complete remission of neuroblastoma in 3 out of 11 pediatric patients (3). These data indicated that a single activating signal mediated by the TCRζ chain is not sufficient to obtain a full activation of T cells as far as persistence, cytokine release, and proliferation is concerned (4, 5). To overcome the first-generation CAR-T cell limitations, the co-stimulatory endodomain of CD28 molecule was added to the intracellular tail of CD3ζ-CARs (6); these chimeras were referred to as second generation CARs (7) (Figure 1). Second-generation CARs improved T cell functions by providing T cells with a stronger signal to avoid T cell anergy and apoptosis after antigen binding. The superior activity of the second generation over the first generation CARs was demonstrated *in vitro* and *in vivo* models (8, 9). Preclinical data about the superiority of second generation CAR over the first generation were then corroborated by clinical results (10, 11). In addition, there is evidence that the incorporation of CD28 co-stimulatory domain into CARs may avoid some of the mechanisms that tumor cells utilize to escape from T cells. Indeed, compared to the first generation of CAR T cells, (i) CD28-CAR T cells secrete higher levels of interferon gamma (IFNγ); (ii) efficiently eradicate transforming growth factor beta (TGFβ) producing tumor cells; and (iii) suppress TGFβ inhibition of T cell expansion (12, 13).

**Figure 1:**
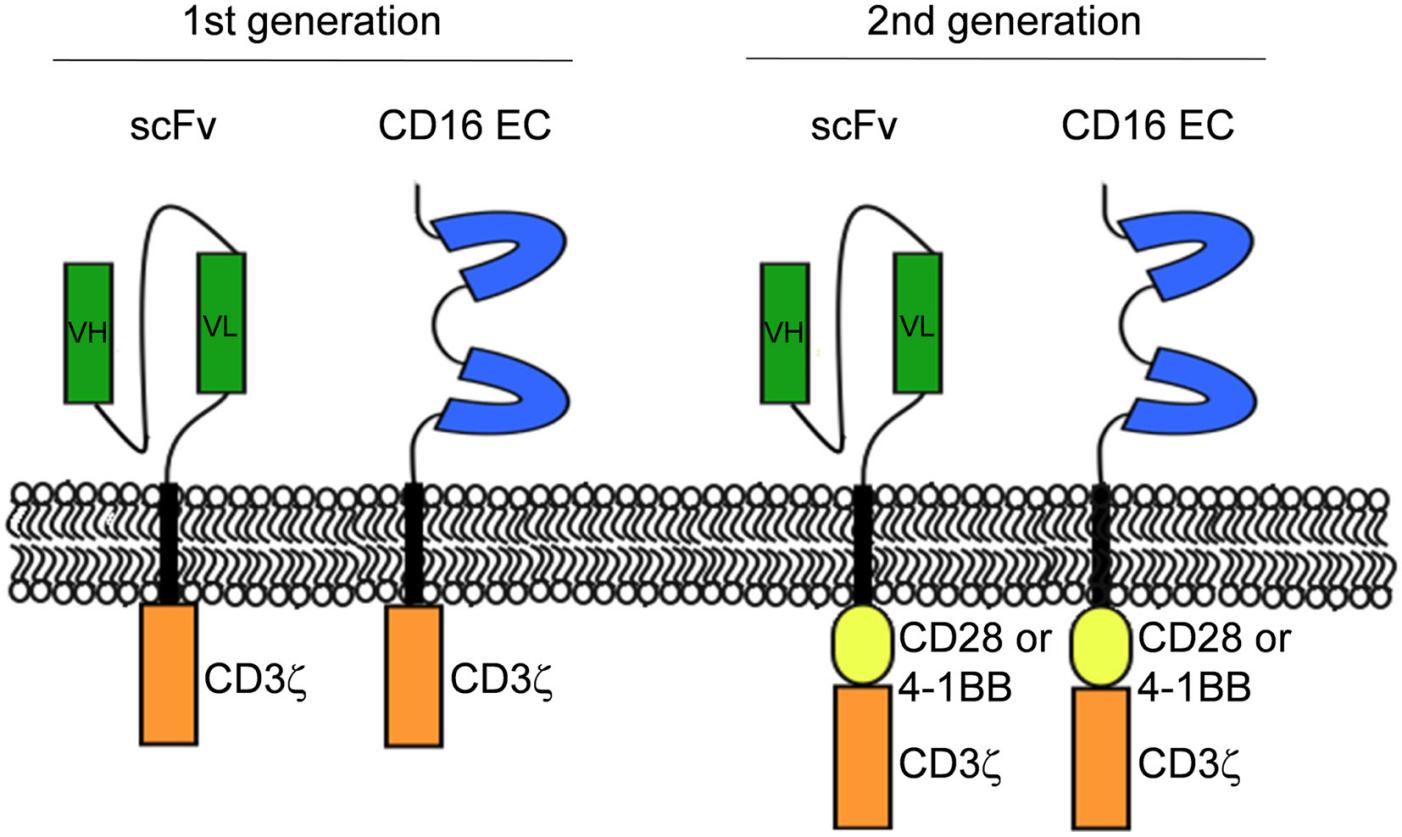
**Schematic representation of CD16-CR and classical chimeric antigen receptor molecular structures**. The first generation of CR has the extracellular domain linked to the intracellular signaling motif of CD3ζ chain while the second generation of CR has an additional co-stimulatory endodomain derived from CD28 or 4-1BB linked to the N-terminal of CD3ζ chain.

The enhancement of T cell activation by the usage of co-stimulatory molecules, into the first generation of CAR was also described by additional studies in which the CD28 molecule was fused in tandem or replaced with 4-1BB (14). Tammana et al. (15) redirected umbilical cord blood T cells to eliminate, *in vitro* and *in vivo*, both lymphoma and leukemia cells by transducing T cells with CD19ζ CAR construct containing CD28 (CD19-28ζ) or 4-1BB (CD19-BBζ) or a combination of both (CD19-28BBζ) co-stimulatory molecules fused with ζ chain in the intracellular domain. They demonstrated that CD19-BBζ CAR T cells and CD19-28BBζ exhibited the highest cytotoxic activity against CD19 positive leukemia and lymphoma cell lines. Interestingly, treatment with CD19-28BBζ prolonged the survival of lymphoma-bearing mice to a greater extent than treatment with CD19-BBζ CAR T cells, indicating that 4-1BB molecule enhances the co-stimulatory properties of the CD28 molecule in umbilical cord blood T cells. In a second study, Song et al. (16) assessed the impact of 4-1BB co-stimulatory signaling on *in vivo* persistence, tumor localization, and antitumor activity of CAR T cells in epithelial cancer. They constructed two CARs containing a folate receptor alpha (FRα) scFv (MOv19) fused with CD3ζ alone (MOv19-ζ) or in combination with the 4-1BB co-stimulatory domain in tandem (MOv19-BBζ). Both MOv19-ζ and MOv19-BBζ CAR T cells secreted proinflammatory cytokines and exerted cytotoxicity in the presence of FRα positive cancer cells *in vitro*. Remarkably, however, only MOv19-BBζ CAR T cells mediated persistent infiltration of the ovarian tumor microenvironment leading to tumor regression in immunodeficient mice (16). Thus, the availability of a second generation CAR which contained a 4-1BB signaling domain resulted in an excellent clinical response and prolonged T cell *in vivo* survival (17). The favorable therapeutic results obtained with the dual-signaling CAR T cells have prompted investigators to hypothesize that the addition of a second co-stimulatory molecule to the CAR would enhance T cells’ antitumor activity. As a result, a third generation CAR composed of two distinct co-stimulatory endodomains was designed. Different combinations of co-stimulatory proteins (e.g. 4-1BB/CD28, CD28/OX40) were assessed, showing various effects concerning T cell persistence, cytokine release, and tumor regression (15, 18).

More recent investigations aimed to boost the therapeutic potential of CAR T cell technology have focused on the development of strategies to arm CAR T cells with tools to counteract immunosuppression mechanism(s) present in the tumor microenvironment. Several strategies have been employed to prevent CAR T cell depletion by tumors such as genetic modifications to express pro-inflammatory cytokines including interleukin-12 (IL-12) and interleukin-15, chemokine receptors or co-stimulatory ligands (19). CD19-CAR T cells genetically engineered to express IL-12 transgene demonstrated improved survival, stronger cytotoxic function, and significant resistance to Treg inhibition. Furthermore, they favored modulation of tumor-associated immune cells, resulting in high tumor eradication in mouse models (20). In the same way, the CD19-CAR T cells genetically engineered to express CD40L, a tumor necrosis factor superfamily member, displayed enhanced antitumor efficacy against CD40^+^ cancer cells, associated with their ability to affect the tumor phenotype by increasing tumor cell sensitivity to Fas-dependent apoptosis and immune destruction (21).

The high antitumor activity displayed by adoptive CAR T cell transfer, during early clinical trials in B cell malignancies, represents a point of strength of this technology. In contrast, CAR T cell-based immunotherapy has demonstrated a limited efficacy with solid tumors. On the other hand, a limitation of this strategy is represented by the reported on-target and off-target toxicity (22). Side effects as cytokine release syndrome and prolonged B-cell depletion were associated with CD19 CAR-T cells infusion (23). The extended *in vivo* persistence of T cell transfer may be advantageous as far as anticancer efficacy is concerned but may be harmful to the host. Although many strategies have been implemented to reduce adverse events, such as the introduction of inducible suicide genes (24), interventions aimed to improve CAR-T cell safety remain a priority.

Then, additional therapeutic strategies designed to implement CAR T cell functions but capable of controlling their toxicity may be underway. One of these strategies involves the Fcγ-CRs that are atypical CAR composed of the Fcγ receptor extracellular domain fused to the intracellular signaling motif of CD3ζ [Fc gamma chimeric receptor (Fcγ-CR)]. A typical example is the extracellular FcγIIIA (CD16)-CR. CD16-CR diverges from the typical CAR structure, due to the replacement of the scFv, which recognizes the targeted TAA with the extracellular region of the CD16. In this context, we will first describe the characteristics of CD16-CRs, and then we will discuss their application in tumor immunotherapy.

## Therapeutic Monoclonal Antibodies (mAbs) and FcγRs

In the last decades, steady progress has been made in human cancer treatment with the introduction of therapeutic TAA-specific mAbs. Rituximab (anti-CD20 mAb), trastuzumab (anti-Her2/neu mAb), and cetuximab [anti-epidermal growth factor receptor (EGFR) mAb] represent just some examples of mAbs with demonstrated efficacy in the treatment of B-cell non-Hodgkin lymphoma (NHL), Her2^+^ breast cancers, colorectal carcinoma, and head and neck cancers, respectively (25, 26). Recently, many efforts have been made to characterize the mechanisms of action that underlie the clinical success of therapeutic TAA-specific mAbs. Convincing evidence indicates that mAbs exert their antitumor effect by two mechanisms. The first is due to the mAb’s ability to interfere with molecular signals involved in malignant cell growth and survival, leading to a direct cell death. The second is immunologic. It is mediated by antibody-dependent cellular cytotoxicity (ADCC) in which mAbs trigger the targeting of cancer cells by innate immune effector cells mainly natural killer (NK) cells (25) and induce or enhance TAA-specific immunity by cognate T cells (27).

The FcγRs are a family of surface proteins composed of three classes: FcγRI (CD64), FcγRII (CD32), and FcγRIII (CD16) with similar structures but distinct functions. They are widely distributed on the surface of immune cells like NK cells, monocytes, macrophages, dendritic cells (DCs), and B cells (28). When the antibody engages the CD16 on NK cells, it triggers downstream activating pathways resulting in perforin/granzyme-dependent tumor target cell lysis (29). Several lines of evidence suggest the involvement of the FcγRs in the antitumor activity of therapeutic mAbs. They include the lower antitumor activity of rituximab and trastuzumab in mice lacking FcγR compared to wild-type mice (30), and the association between CD16 genetic polymorphism with patients’ clinical responses to mAb treatment observed in some malignant diseases (31, 32). Notably, the presence of CD16 158-valine allelic polymorphism predicted a better clinical response to rituximab treatment in NHL patients and an improved clinical outcome in colorectal carcinoma patients treated with cetuximab (32). So, evidence coming both from preclinical and clinical studies underlie the high impact of ADCC and FcγRs on mAb anticancer activity. A list of the therapeutic mAbs with demonstrated ADCC activity is reported in Table 1.

**Table 1:** **Summary of therapeutic monoclonal antibodies (mAbs) with proved antibody-dependent cellular cytotoxicity (ADCC) activity**.

Name	Target disease	Target antigen	Immunoglobulin G (IgG) subclasses	Type
Rituximab (Rituxan)	Non-Hodgkin lymphoma (NHL)	CD20	IgG1	Chimeric
Ofatumumab (Arzerra)	Chronic lymphocytic leukemia (CLL)	CD20	IgG1	Human
Ocaratuzumab	CLL	CD20	IgG1	Humanized
Tositumomab (Bexxar)	NHL	CD20	IgG2	Murine
Lucatumumab	Relapsed CLL	CD40	IgG1	Murine
	Multiple myeloma (MM), NHL, and HL			
Daratumumab (Darzalex)	MM	CD38	IgG1	Human
Alemtuzumab (Campath-1H)	CLL	CD52	IgG1	Humanized
Cetuximab (Erbitux)	Squamous cell carcinoma	Epidermal growth factor receptor (EGFR)	IgG1	Chimeric
	CRC			
Panitumumab (Vectibix)	CRC	EGFR	IgG2^a^	Human
Trastuzumab (Herceptin)	BC	ErbB2	IgG1	Humanized
	HER2^+^			
Avelumab	Bladder cancer, gastric cancer, mesothelioma, non-small-cell lung carcinoma, ovarian cancer, head and neck cancer, renal cell carcinoma	PDL-1	IgG1	Human
Mogamulizumab	Adult T cell leukemia, peripheral T-cell lymphoma	CC chemokine receptor 4	IgG1	Humanized

*^a^An IgG_2_ subclass of mAb capable of mediating ADCC by myeloid cells only*.

The ADCC-mediated clinical success of mAbs is influenced not only by the CD16 allotypes but also by tumor accessibility to cytotoxic cells. In some cases, the tissue architecture may be difficult to reach by the ADCC effector cells such as NK cells, which poorly infiltrate tumor microenvironment (33). In these cases, the ADCC-mediated anticancer activity of mAbs is reduced. A novel approach useful to overcome this NK cell limitation and to enhance mAbs’ cell-mediated cytotoxicity utilizes Fcγ-CR-engineered T cells since CTLs easily infiltrate the tumor microenvironment and their presence is closely associated with a favorable course of the disease in many types of cancers (34).

## CD16-CRs

**Figure 2:**
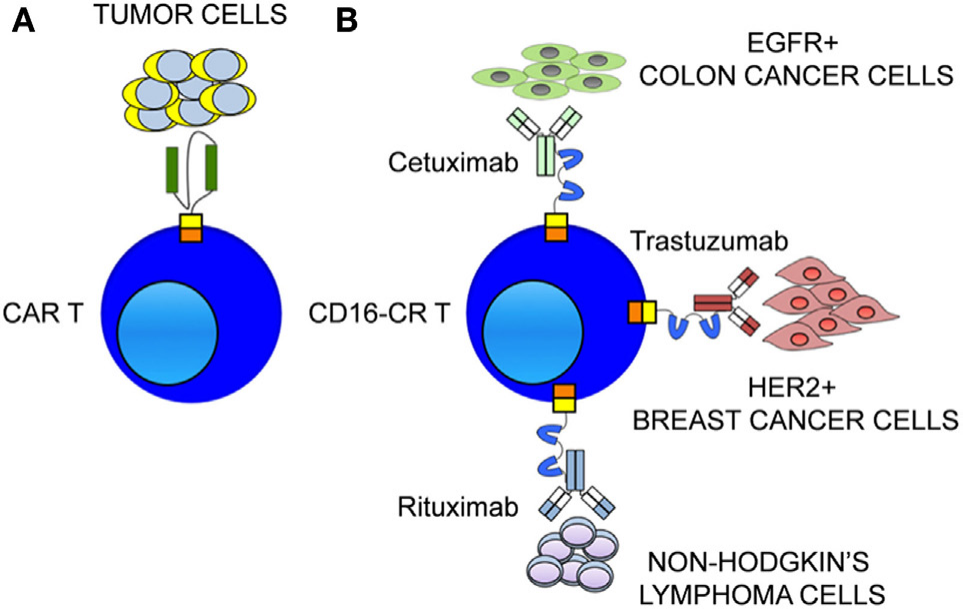
**Therapeutic monoclonal antibodies (mAbs) confer to CD16-CR T cells multiple target specificities**. **(A)** The ability of chimeric antigen receptor (CAR) to redirect T cells toward tumor cells is restricted to a single tumor-associated antigen (TAA) expressed on tumor cell surface. **(B)** The availability of therapeutic mAbs allows CD16-CR to redirect T cells virtually against all TAA expressed on a variety tumor types including hematological and epithelial malignancies.

Two generations of CD16-CRs have been reported (35–38) (Figure 1). T cells transduced with CD16-CR display antitumor activity only when they are combined with mAbs. Preclinical studies have demonstrated that CD16-CRs trigger cell-mediated cytotoxicity against lymphoma cell lines opsonized with the CD20-specific mAb, rituximab. However, no clinical trials have been conducted to date (Table 2). CD16-CR T cells share with CAR T cells advantages and disadvantages. The former include the ability to mediate an HLA-unrestricted cell-mediated cytotoxicity leading to the elimination of cancer cells with abnormalities in HLA-class I antigen processing machinery component expression and/or function. The latter involve the targeting of a TAA also expressed in normal tissues. However, this atypical CAR brings also three additional advantages compared to classical CARs, although not yet demonstrated. The first one is the possibility to target different types of tumor cells provided that the TAA-specific mAb with the required specificity are available (Figure 2). The second one is the possibility to improve control of their off-target toxicity, simply by eliminating the supply of mAbs to CD16-CR T cells through mAb dosage tapering or termination during patient treatment. The third one is the opportunity to mitigate acute toxicity by the administration of high-doses of immunoglobulins. On the other hand, CD16-CR T cells bear additional limitations since therapeutic mAbs may compete with serum immunoglobulins for their binding to the Fcγ CRs and may be defective in their ability to mediate ADCC. Other potential disadvantages of Fcγ-CR T cells could be hypothesized when these cells are given to patients with high levels of autoantibodies or viral-specific antibodies following an infectious episode. In the course of autoimmunity, autoantibodies may redirect engineered T cells against self-antigens. In contrast during an infectious episode, specific-antivirus antibodies could redirect the virus toward engineered T cells, by the Fc fragments, favoring a viral infection of the cells, which may lead to T cell aberration (39).

**Table 2:** **Summary of published preclinical studies involving CD16-CR-engineered T cell-based immunotherapy**.

CD16-CRs	Structure	Malignant cells	Associated monoclonal antibody	Reference
CD16/γ	CD16 (EC) + FcεRIγ [transmembrane (TM), IC]	B-lymphoblastoid	Rituximab	Clémenceau et al. (35)
CD16ζ	CD16(EC) + CD3ζ (TM, IC)	CD20^+^ lymphoma, HER2/neu^+^ breast cancer, and T cell leukemia	Rituximab	Ochi et al. (36)
			Trastuzumab	
			Mogamulizumab	
CD16V-BB-ζ	CD16(EC) + CD8a (TM) + 4-1BB + CD3ζ (IC)	CD20^+^, primary B chronic lymphocytic leukemia, neuroblastoma	Rituximab	Kudo et al. (37)
			Hu14.198K322A	
CD16-28-ζ	CD16(EC) + CD8a (TM) + CD28 + CD3ζ (IC)	Burkitt’s lymphoma	Rituximab	D’Aloia et al. (38)

## First Generation of CD16-CR

With the goal to utilize cell-mediated cytotoxicity as a mechanism to potentiate the anticancer activity of TAA-specific mAb, Clémenceau and colleagues (35) proposed to engineer T lymphocytes with the first generation of a CD16-CR. They developed a fusion protein composed of the extracellular domain of CD16 ligated to the transmembrane (TM) and the intracellular domain of FcεRIγ (CD16/γ). This construct was utilized to transduce TCRαβ CD4^+^ and CD8^+^ HLA-DPB1*0401-specific T cells. The cytotoxic activity of the CD16/γ-T cells was demonstrated *in vitro* against HLA-DPB1*0401-positive, and negative B-lymphoblastoid cell lines opsonized with rituximab. Target cell lysis was detected neither with non-transduced T cells against HLA-DPB1*0401-negative cell lines nor with CD16/γ-transduced T cells without rituximab. Conversely, in the presence of rituximab, both HLA-DPB1*0401-positive and negative cell lines were killed at a similar level by transduced T lymphocytes. Overall, these results indicated the ability of the CD16/γ receptor to trigger T cell cytotoxicity against mAb-opsonized target cells without prior TCR recognition. Furthermore, transduced T cell clones showed active proliferation and cytokine release upon CD16 crosslinking by mAb-coated target cells. Epstein–Barr virus-specific CTLs were also transduced with the CD16/γ-CR and were used to confirm that CD16/γ-CR expression confers the ability to mediate ADCC both to CD4^+^ and CD8^+^ T cells.

The *in vitro* characterization of the CD16/γ -T cells performed by Clémenceau et al. (35) for the first time demonstrated the possibility to transfer the ADCC capacity to T cells. With a similar purpose, later Ochi and collaborators (36) proposed a variant of the CD16-CR obtained by linking the CD16 with the TM and the intracellular portion of CD3ζ (CD16ζ). The chimeric receptor was successfully expressed by lentiviral transduction on T cell surface of a healthy donor, and ADCC activity was reported against CD20^+^ lymphoma, Her2/neu^+^ breast cancer and T-cell leukemia cell lines coated with rituximab, trastuzumab, and mogamulizumab (Mog), respectively. The tumoricidal activity of CD16ζ T cells was increased by enhancing the dose of the mAb and was blocked by a CD16-specific mAb termed 3G8. Following the CD16ζ engagement by the mAb-opsonized cancer cells, CD16ζ-transduced T cells displayed several functional activities such as IFNγ and IL-2 secretion, lytic granule release, and proliferation. Furthermore, Ochi and colleagues assessed the antitumor activity of the CD16ζ T cells generated through the transduction of peripheral blood mononuclear cells harvested from CD20^+^ B-cell lymphoma patients. They found that CD16/γ-T cells exerted anti-tumor activity against autologous tumor cells to the same extent of healthy donors’ transduced T cells.

The antitumor efficacy of CD16ζ T cells combined with the specific therapeutic mAb was also validated *in vivo*. A tumor growth inhibition was reported in B-cell lymphomas immunodeficient xenografted murine model when mice were treated with a combination of rituximab and CD16ζ T cells. Conversely, no significant tumor mass reduction was observed in mice treated with CD16ζ T cells or rituximab alone compared to untreated animals. The overall survival reflected the same pattern: the regimen of CD16ζ T cells plus rituximab prolonged mice survival to a significantly greater extent than other treatments. The authors emphasized that CD16ζ T cells have a better *in vivo* anti-lymphoma activity than NK cells (36).

Recently, an anti-adult T cell leukemia (ATL) effect has also been reported utilizing CD16ζ T cells in association with Mog, an anti-CC chemokine receptor 4 mAb (40). T cells deriving both from healthy donors and ATL patients armed with CD16ζ-CR exerted a significant ADCC against Mog-coated leukemia cell lines and ATL primary cells. Furthermore, the simultaneous infusion of Mog and CD16ζ T cells in ATL xenografted mouse models significantly blocked tumor spread and prolonged mice survival.

Despite the encouraging preclinical results obtained both with the CD16/γ and the CD16ζ first-generation CRs, no clinical trials have been conducted yet.

## Second Generation of CD16-CR

The second generation of CD16-CR was described both by Kudo et al. (37) and by D’Aloia et al. (38). Kudo et al. generated the CD16V-BB-ζ-CR by introducing the TM portion of CD8a and the co-stimulatory endodomain of the 4-1BB into the module of the first-generation CD16ζ-CR (37). After CD16V-BB-ζ transduction into T cells, they investigated the capacity of the CD16-CRs to induce T cell functional activation upon a CD16 receptor crosslinking by an immobilized IgG. They found that CD16V-BB-ζ engagement promoted IL-2 receptor expression and exocytosis of cytotoxic granules by T lymphocytes. The T cells expressing CD16V-BB-ζ -CR were able to mediate granule-dependent ADCC toward CD20^+^ cell lines and primary B chronic lymphocytic leukemia cells. About 50–70% of the target cells were killed in a 4-h co-culture test. The anticancer efficacy of the CD16V-BB-ζ T cells, in the presence of the specific therapeutic mAbs, was also demonstrated against solid tumors such as breast and gastric cancer cell lines. Furthermore, the authors validated the tumor regression capacity of the CD16V-BB-ζ T cells in NOD-SCID IL-2RGnull mice injected with B-cell lymphoma. In all five mice treated with rituximab combined with CD16V-BB-ζ T cells, a stable remission of the tumor mass was observed after 120 days from the injection. Similar results were reported against xenografted neuroblastoma murine model when hu14.198K322A mAb was used with CD16V-BB-ζ T cells.

Furthermore, Kudo et al. compared the CD16V-BB-ζ-CR with the CD16/γ, CD16ζ-CRs, and CD19-CAR demonstrating a superior T cell activation capacity and antitumor activity of the CD16V-BB-ζ (37). They also compared the CD16V-BB-ζ T cells with T cells transduced with a typical CD19-CAR (CD19-BB-ζ) regarding cytotoxic effects. They found that target cell elimination was higher with CD16V-BB-ζ-CR than with CD19-CAR T cells.

**Figure 3:**
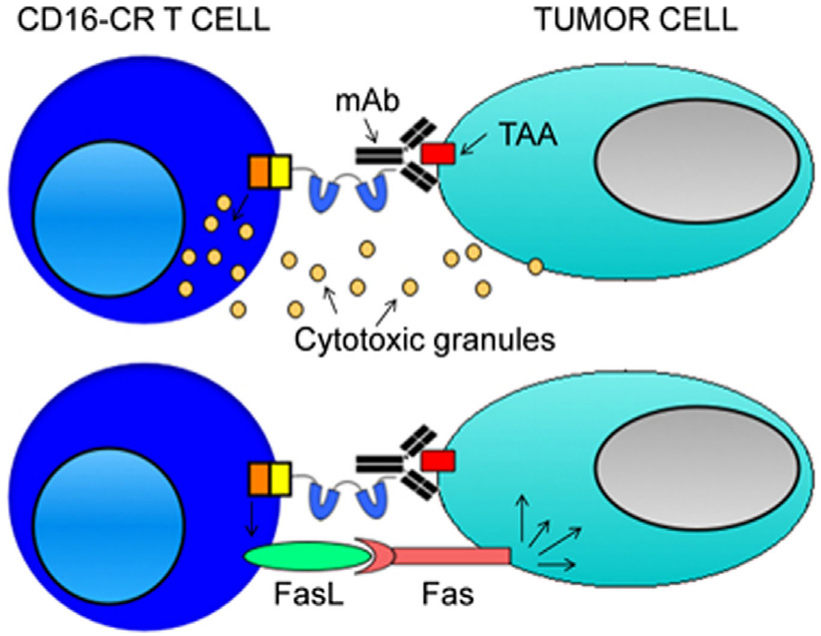
**Mechanisms of CD16-CR T cell-mediated tumor cell elimination**. CD16-CR T cells acquire the specificity of a monoclonal antibody (mAb) recognizing a tumor-associated antigen (TAA) on the cell surface of tumor cells through the binding of the CD16 with the mAb Fc fragment leading to the activation of T cell-mediated cytotoxicity. In this context, engineered T cells kill tumor cells by using two mechanisms. The first involves the activation of T cell killing machinery by the release of cytotoxic granules (upper panel). The second arises from the induction of CD16-CR dependent FAS expression on the cell surface of tumor cells that allows FAS ligand positive-engineered T cells to kill tumor cells by a granule independent cellular cytotoxicity (lower panel). However, the latter mechanism is just a hypothetical model based on a single study employing transformed MD45 mouse T cells. Then, the confirmation of the existence of a role for granule independent cytotoxicity requires additional studies.

While Kudo et al. highlighted the ADCC-mediated antitumor efficacy of the CD16-CR with the co-stimulatory domain of 4-1BB, D’Aloia et al. investigated the ability of a CD16-CR variant carrying the CD28 co-stimulatory motif (CD16-28-ζ) (38). They assessed the functionality of the CD16-CR in the MD45 cell line, a murine T cell hybridoma lacking the lytic granule machinery but with cell killing ability through the Fas/FasL pathway. First, they proved the ability of this chimera to induce MD45 cell activation upon the IgG binding, showing the phosphatidylinositol-3-kinase phosphorylation and IL-2 secretion. The perforin/granzyme deficient MD45 cell line allowed to test the capacity of CD16-CR to trigger tumor cell depletion by the mediation of Fas/FasL pathway. A 16-h cytotoxicity assay performed with Fas^+^ Raji lymphoma cells as target cells demonstrated that about 30% of the tumor cells were lysed by the effector cells, at the highest effector: target ratio, only in the presence of rituximab. A better result regarding the percentage of target cell lysis was reported redirecting MD45-CD16-CR against P815 cells with B73.1, an anti-CD16 mAb, demonstrating that CD16-CR was able to trigger both ADCC and reverse ADCC. Interestingly, the elimination of rituximab-coated Raji cells by MD45-CD16-CR cells was abrogated by an anti-FasL mAb. These results strongly suggest that a Fas/FasL-mediated killing is involved (Figure 3).

## Future Perspectives for Fc-Gamma-CR T Cells

CD16-CR T cells utilized in combination with TAA-specific mAbs have provided clear evidence of antitumor activity toward hematologic and epithelial malignant cells *in vitro* and *in vivo*. The mechanisms by which CD16-CR T cells, in combination with mAbs, eliminate cancer cells involve both granule-dependent and granule independent cellular cytotoxicity. This information represents a platform for developing Fcγ-CR-based targeted therapies of virtually any malignancies, as long as therapeutic mAbs with the appropriate specificity are available. To reach this goal, additional information is needed to optimize Fcγ-CR and mAb combination before testing the described strategy in a clinical setting. To this end, investigators will need to accomplish three primary tasks. The first task will be aimed to identify strategies that will increase the pool of therapeutic mAbs capable of redirecting T cells against cancer cells. The second task should have the objective to determine the best solid tumor to be targeted. The third task should determine *in vivo* toxicity of Fcγ-CR-based immunotherapy.

In this context, it is critical to consider the basis of the interaction of therapeutic mAbs with Fc receptors. The isotype IgG1 is the prevalent subclass of IgG mAbs utilized in the clinic (Table 1). IgG1 mAbs preferentially bind CD16^valine-158-valine^ but also CD16^valine-158-phenylalanine^ variants. They are capable of triggering NK cell and monocyte-mediated ADCC but also antibody-dependent cellular phagocytosis, and complement-dependent cytotoxicity. In contrast, IgG2 is also used in mAb therapeutics; however, it elicits significant weaker ADCC since it does not trigger NK cell-mediated cytotoxicity, as compared to IgG1. Nevertheless, they are still capable of eliciting a myeloid cell-mediated cytotoxicity (41). As a consequence, the use of IgG2 therapeutic mAbs in EGFR positive malignancies, and particularly in those with KRAS mutations, may trigger lower antitumor activity than IgG1 mAbs. Interestingly, most subclasses of monomeric mAbs including IgG1, IgG3, and IgG4 but not IgG2 display a high-affinity Fc binding to the FcγRI (CD64) leading to the stimulation of a potent myeloid-mediated cytotoxicity and phagocytosis. Unfortunately, CD64-mediated ADCC may have a limited impact on the inhibition of tumor progression since it does not involve NK cells. Given the availability of four IgG2 and three IgG4 therapeutic mAbs, it is likely that investigators will maximize the antitumor effect of the therapeutic mAb of interest by engineering T cells with the most appropriated Fcγ-CR. Since, the CD16-CR is the only molecule today available in the laboratories, in order to identify the best combination of therapeutic mAb with Fcγ-CR, there will be a need for developing the CD32 and CD64 CRs to be utilized in additional studies *in vitro* and *in vivo*.

It is noteworthy that solid tumors are not an ideal target for immune cells. This is mainly due to the ability of cancer cells to avoid immune cells by utilizing several types of escape mechanisms affecting accessibility, persistence, and function of immune cells in the tumor microenvironment. Compelling evidence suggests that solid tumors do not promote an efficient ADCC since their microenvironment is deficient in NK cells (42) but rich in M2 macrophages endowed with immunosuppressive and pro-angiogenic functions (43) and regulatory T cells (44). As a consequence, such an anti-inflammatory microenvironment favors tumor progression. Nevertheless, colorectal carcinoma (CRC) represents an interesting exception. Among solid tumors, immune cell infiltration in the CRC microenvironment is associated with improved overall survival even in the presence of known immunosuppressive cells such as tumor-associated macrophages (45, 46), and regulatory T cells (44). In addition, two lines of evidence suggest that ADCC contributes to the antitumor activity of anti-EGFR mAbs. First, a subset of CRC cells is consistently infiltrated by NK cells (47, 48). Second, the presence of a CD16^valine-158-valine^ predicts favorable clinical responses in CRC patients (32). Based on this information in our opinion, CRC could be an ideal target for assessing the antitumor activity of Fcγ-CR T cells.

Finally, to determine the potential toxicity of this treatment, it may be useful to utilize immunocompetent mice bearing a spontaneous or engrafted CRC to be targeted with mouse Fcγ-CR T cells in combination with mouse anti-EGFRs mAbs.

## Author Contributions

SC, SF, and GS conceived and wrote the manuscript. TS, RA, AC, GL, DL, AV, MIDP, FB, LM, and MP have critically reviewed and revised the manuscript. All authors fully agreed with the manuscript content.

## Conflict of Interest Statement

The authors declare that the research was conducted in the absence of any commercial or financial relationships that could be construed as a potential conflict of interest.

## Abbreviations

ADCC, antibody-dependent cellular cytotoxicity; ADCP, antibody-dependent cellular phagocytosis; ATL, adult T cell leukemia; BC, breast cancer; BLCL, B lymphoblastoid cell line; CAR, chimeric antigen receptor; CCR4, CC chemokine receptor 4; CDC, complement-dependent cytotoxicity; CLL, chronic lymphocytic leukemia; CRC, colorectal carcinoma; CTL, cytotoxic T cell; DC, dendritic cell; EC, extracellular; EGFR, epidermal growth factor receptor; Fcγ-CR, Fc gamma chimeric receptor; FRα, folate receptor alpha; HL, Hodgkin lymphoma; HN, head and neck; IC, intracellular; IFNγ, interferon gamma; IgG, immunoglobulin G; IL-12, interleukin-12; IL-15, interleukin-15; mAb, monoclonal antibody; MHC, major histocompatibility complex; MM, multiple myeloma; Mog, mogamulizumab; NHL, non-Hodgkin lymphoma; NK, natural killer; NSCLC, non-small-cell lung carcinoma; OC, ovarian cancer; PBMC, peripheral blood mononuclear cell; PTCL, peripheral T-cell lymphoma; RCC, renal cell carcinoma; scFv, single-chain variable fragment; TAA, tumor-associated antigen; TCR, T cell receptor; TGFβ, transforming growth factor beta; TM, transmembrane.
